# A land classification protocol for pollinator ecology research: An urbanization case study

**DOI:** 10.1002/ece3.4087

**Published:** 2018-04-30

**Authors:** Ash E. Samuelson, Ellouise Leadbeater

**Affiliations:** ^1^ School of Biological Sciences Royal Holloway University of London Egham UK

**Keywords:** agricultural pest control, anthropogenic stressors, bees, GIS, land classification, land‐use change, pollinator, urbanization

## Abstract

Land‐use change is one of the most important drivers of widespread declines in pollinator populations. Comprehensive quantitative methods for land classification are critical to understanding these effects, but co‐option of existing human‐focussed land classifications is often inappropriate for pollinator research. Here, we present a flexible GIS‐based land classification protocol for pollinator research using a bottom‐up approach driven by reference to pollinator ecology, with urbanization as a case study. Our multistep method involves manually generating land cover maps at multiple biologically relevant radii surrounding study sites using GIS, with a focus on identifying land cover types that have a specific relevance to pollinators. This is followed by a three‐step refinement process using statistical tools: (i) definition of land‐use categories, (ii) principal components analysis on the categories, and (iii) cluster analysis to generate a categorical land‐use variable for use in subsequent analysis. Model selection is then used to determine the appropriate spatial scale for analysis. We demonstrate an application of our protocol using a case study of 38 sites across a gradient of urbanization in South‐East England. In our case study, the land classification generated a categorical land‐use variable at each of four radii based on the clustering of sites with different degrees of urbanization, open land, and flower‐rich habitat. Studies of land‐use effects on pollinators have historically employed a wide array of land classification techniques from descriptive and qualitative to complex and quantitative. We suggest that land‐use studies in pollinator ecology should broadly adopt GIS‐based multistep land classification techniques to enable robust analysis and aid comparative research. Our protocol offers a customizable approach that combines specific relevance to pollinator research with the potential for application to a wide range of ecological questions, including agroecological studies of pest control.

## 1 INTRODUCTION

A large body of evidence suggests that insect pollinators, including bees, are under threat (Biesmeijer et al., 2006; Potts et al., 2010). Multiple anthropogenic drivers have been identified (Goulson, Nicholls, Botías, & Rotheray, 2015), with land‐use change and the associated loss of habitat proposed as one of the most critical threats (Potts et al., 2015). Strong negative effects of landscape alteration on bee and wasp species richness and composition have been documented (Senapathi et al., 2015), with habitat‐ and food‐specialist pollinator taxa particularly vulnerable (González‐Varo et al., 2013). However, the impacts of land‐use on different aspects of pollinator ecology and on different pollinator taxa can be complex, with effects varying depending on pollinators’ dietary and dispersal strategies (Steffan‐Dewenter, Münzenberg, Bürger, Thies, & Tscharntke, 2002; Winfree, Aguilar, Vázquez, Lebuhn, & Aizen, 2009) and the type and magnitude of the land‐use change in question (Cariveau & Winfree, 2015; Senapathi, Goddard, Kunin, & Baldock, 2017). As a result, the impact of land‐use change on pollinator populations remains a considerable knowledge gap.

Comprehensive quantitative methods for classifying the land surrounding study sites are critical to producing a robust analysis of the effects of land use (Owen et al., 2006). The more rigorous the land classification, the greater the flexibility of the questions that can be asked about its effects, and the less subjective the interpretation of land‐use types. In the pollinator literature, methods used vary widely, and there has historically been no single commonly adopted land classification approach. Broadly, the approaches used can be grouped into three categories: (i) simple visual classification; (ii) geographical information system (GIS)‐based single‐step classification; and (iii) GIS‐based refined classification. The former typically involves locating study sites in extreme and/or representative examples of land‐use types (e.g., nature reserve, agricultural land, city) and using these qualitatively defined types as a categorical land‐use variable, often associated with qualitative descriptions of features of the land‐use types but with no further analysis (e.g., Banaszak‐Cibicka, Fliszkiewicz, Langowska, & Żmihorski, 2017; Goulson, Hughes, Derwent, & Stout, 2002). GIS‐based single‐step classification typically employs a more quantitative approach, using unmanipulated variables directly extracted from existing data layers or remote‐sensing data such as “proportion impervious surface” or “proportion agricultural land” as defined by the classification system of the data layer in question (e.g., Williams, Regetz, & Kremen, 2012; Youngsteadt, Appler, López‐Uribe, Tarpy, & Frank, 2015) or a combination of a number of these variables (e.g., Baldock et al., 2015; Donkersley, Rhodes, Pickup, Jones, & Wilson, 2014; Senapathi et al., 2015). These variables may be categorized by nonstatistically defining criteria, *for example*, “Agricultural = More than 50% of the surrounding designated landscape composed of agricultural areas” (Lecocq, Kryger, Vejsnæs, & Jensen, 2015). Finally, GIS‐based refined classification involves an additional step or steps to manipulate combinations of relevant land variables into a smaller number of variables containing the same information using statistical tools (e.g., Sponsler & Johnson, 2015; Verboven, Uyttenbroeck, Brys, & Hermy, 2014). This type of approach typically affords more capability to generate a land classification tailored to the study question, as we will argue below.

As land classification methods have advanced, there has been a slow shift within the field of pollinator ecology toward adopting the latter approach. However, uptake has been far from universal and land classification protocols are typically less powerful than those currently in common use in geographical disciplines. A reasonable criticism of land classification in pollinator studies is that using land‐use variables that have been developed from a human perspective, such as proportion urban land as defined by a topographic mapping data layer, can be an ill fit for the aspects of the landscape that are relevant to pollinators (Senapathi et al., 2017). For example, urban land consisting of residential houses and gardens may represent a considerably richer habitat for bees than an industrial estate or central business district (Foster, Bennett, & Sparks, 2017), or agricultural areas growing flowering crops may be richer than those growing cereals (Riedinger, Mitesser, Hovestadt, Steffan‐Dewenter, & Holzschuh, 2015). This information may be lost in extracting data from existing classifications, particularly if demographic variables such as human population density are used (Matteson, Grace, & Minor, 2013). In essence, it can be argued that adopting human‐focussed land classification for pollinator research is at best a proxy for land classification from the pollinator's perspective.

Techniques for generating a land classification from raw data as a bottom‐up approach can draw on existing methods used in geographical disciplines (Hahs & McDonnell, 2006; Owen et al., 2006) and allow flexibility in adapting the land classification to the specific research question. For example, in studies where transient land cover information is required, such as crops grown and bloom stage, data from ground surveys may be incorporated into the land classification. The resolution of the land classification can also be tailored to the space use of the taxon in question; available land cover data layers are often at resolutions too low to be appropriate for the resolution at which pollinators interact with the land (Büttner et al., 2004). A bottom‐up approach also allows extraction of multiple land‐use variables at different levels of categorization. For example, the question “how does agricultural land‐use affect pollinator abundance?” may be followed up by investigating whether any effect found is driven by the extent of wildflower strips in the surrounding area. The spatial scale at which a pollinator responds to the surrounding land depends on its space use (e.g., foraging range) and the response variable in question (e.g., relating to nesting, foraging, or mating behavior) (Steffan‐Dewenter et al., 2002; Westphal, Steffan‐Dewenter, & Tscharntke, 2006); a pollinator‐focussed land classification protocol can include data‐driven methods for assessing this.

In this study, we develop a flexible approach to land classification that is appropriate for research into the effects of land use on pollinators, using urbanization as an example. The advantages of a bottom‐up approach are particularly apparent for urban land classification, as its high level of heterogeneity at a fine resolution is often missed with coarser classification methods, and its typically intransient land cover patches are well suited to visual classification from satellite imagery. Urban ecology is a growing field (Adams, 2005), and in recent years, attention has begun to focus on the effects of urbanization on pollinators (Baldock et al., 2015; Harrison & Winfree, 2015). The wide array of land classification techniques that have been employed in this growing body of literature can make comparisons between studies difficult, generating a call for wider adoption of geographical approaches (Winfree, Bartomeus, & Cariveau, 2011).

**Figure 1:**
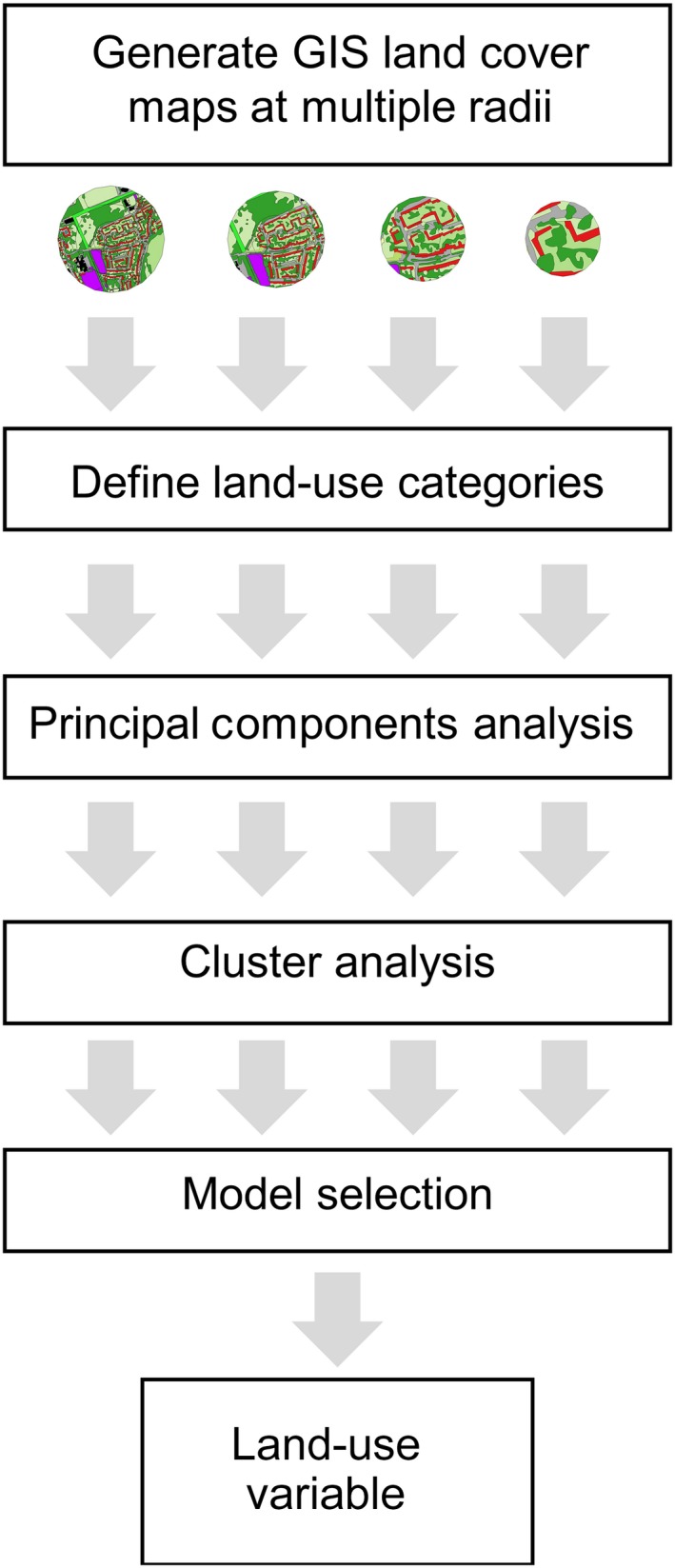
Overview of the multistep protocol presented for land classification in pollinator ecology research

The protocol that we present combines primary land cover classification using GIS with a focus on identifying land cover types that have a specific relevance to bees and other pollinators, followed by information refinement using statistical tools (Figure 1). Refinement consists of a three‐step process: (i) definition of land‐use categories, (ii) principal components analysis (PCA) on the categories, and (iii) cluster analysis to generate a categorical land‐use variable for use in subsequent analysis. We present a case study for land classification of 38 sites in South‐East England across a gradient of urbanization, within which bumblebee colonies were placed for a study investigating the effects of urban land use on colony success.

## 2 METHODS

### 2.1 Study area

**Figure 2:**
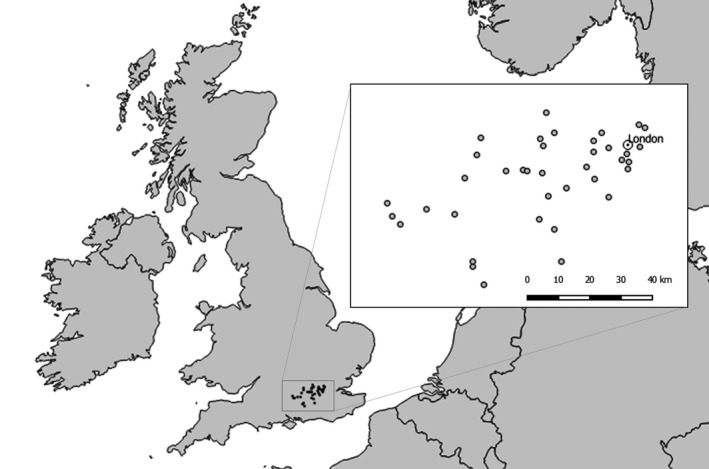
Location of 38 sites in SE England for which land classification was carried out using the protocol presented here

Thirty‐eight sites were located across a c. 5,000 km^2^ area in SE England (Figure 2) spanning an urbanization gradient from dense continuous urban development in central London (most easterly site: 51°32′59.5644″N, 0°2′25.3284″W) to agricultural land in the counties of Hampshire, Surrey, and Berkshire (most westerly site: 51°20′17.1096″N, 1°12′24.9469″W). This represents a typical urbanization gradient in western Europe, with dense urban land transitioning into a wide suburban belt before giving way to agriculture.

### 2.2 Creating a land cover map

For the purposes of this study, we use the term land cover to refer to surface cover and land use to refer to data generated from land classification containing information about various aspects of the land. Our protocol involves manual generation of a land cover map based on visual inspection rather than using existing data layers to increase flexibility in selecting resolution, allow later combination with ground survey data, and increase relevance to pollinator‐specific use of landscape through discrimination of relevant habitats (e.g., gardens or wildflower strips). Sites were located using Google Earth (version 7.1.5.1557) by navigating to the nearest postcode and visually adding a Placemark at the exact location of colony placement at an “eye altitude” of 500 m. The site locations were imported as a.kml file into QGIS version 2.16 and saved as a.shp file for manipulation as a data layer in QGIS. The sites data layer was overlaid onto the Web‐based satellite imagery layer Bing Aerial from the OpenLayers plugin (http://www.openlayers.org). A 750‐m circular buffer [the largest spatial scale of four selected for the land classification (see below), based on *B. terrestris* typical foraging range (Darvill, Knight, & Goulson, 2004; Knight et al., 2005; Osborne et al., 1999)] was generated around each site with a separate data layer for each site.

**Figure 3:**
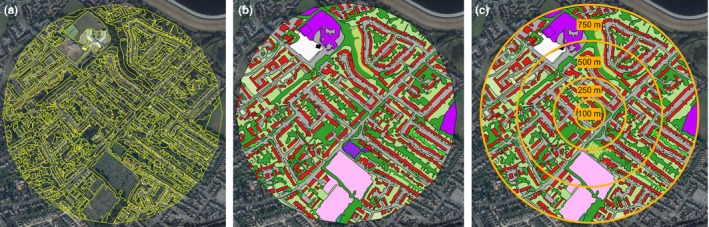
Illustration of the steps involved in manually generating a land cover map for a 750 m radius around a study site in QGIS, using an example site in the suburban region to the southwest of London, UK. (a) The first step involves drawing polygons around each land cover patch at a set scale (1:5,000 m in agricultural areas or 1:2,500 m in built‐up areas) to split the data layer into a series of features representing each patch. (b) Each patch is visually classified to one of 80 land cover classes; for color legend, see Appendix S1. (c) The buffer is clipped to multiple radii representing different spatial scales at which the study taxon may interact with the surrounding land based on ecology of the organism

Land cover patches were classified within the buffers surrounding each site. At a scale of 1:5,000 m in agricultural areas, or 1:2,500 m in built‐up areas, polygons were drawn around each land cover patch using the QGIS “Split Features” and “Fill Ring” tools, separating the buffer layer into a series of features representing individual patches of a single land cover type, at a resolution separating individual buildings (or joined sets of buildings), fields, and gardens (Figure 3a). The resolution at which patches are separated may be adapted to the focus of the study; for example, it may be more appropriate to group areas of similar density of urban development rather than separating individual buildings for a honeybee study, due to the greater foraging range of honeybees. Each polygon was visually assigned to one of 34 initial land cover classes (e.g., house, residential garden, arable field, hedgerow; for full list, see Appendix S1) by entering a two‐letter code in the “Description” field of the attribute table. For visualization purposes, a layer style was generated with a color assigned to each land cover class (Figure 3b).

### 2.3 Maps at multiple radii

The spatial scale at which pollinators respond to the surrounding landscape varies depending on aspects of behavior and ecology such as foraging range and the response variable in question (Steffan‐Dewenter et al., 2002; Westphal et al., 2006). Land cover maps at multiple biologically relevant radii may therefore be generated for later comparison using model selection techniques (see below). In addition to the 750‐m buffer, buffers of 500, 250, and 100 m [representing steps of spatial scales at which bees may interact with the surrounding land (Carvell et al., 2017; Moreira, Boscolo, & Viana, 2015)] were added by clipping the initial buffer layer to generate new data layers at the specified radii. Each site thus had four associated land cover map layers (Figure 3c).

### 2.4 Ground surveys

Visually classifying land cover using satellite imagery is suitable for intransient land cover types such as urban or water body land classes, but not for transient land cover classes such as crops because readily available satellite imagery is typically not updated annually. In addition, crops may not be imaged during their flowering period, making them unidentifiable from satellite imagery. It is therefore recommended to supplement GIS classification with ground surveys to produce an up‐to‐date picture of the land use at the time of the study. This is particularly important for bee research, as bees may forage on floral resources such as oilseed rape, which are highly transient between seasons (Riedinger et al., 2015).

Ground surveys were carried out in May 2016, while bumblebee colonies were in the field, at all sites which contained agricultural land within a 750 m radius (*n* = 19). For each site, agricultural fields were visited by car or on foot, and the crop grown, bloom stage, and presence of wildflower strips and other floral resources recorded. This information was incorporated into the existing GIS, splitting polygons where necessary to add wildflower strips. This resulted in a total of 80 land cover classes.

### 2.5 Defining land‐use variables from the pollinator's perspective

Eight land‐use categories were defined: impervious surface (including buildings), flower‐rich habitat, domestic infrastructure (including parks), gardens, tree cover, agricultural land, open land, and road (excluding vegetated verges). These groupings were developed by considering land‐use factors that bees are likely to respond to based on foraging and nesting ecology. Each of the 80 land cover classes was coded according to whether it belonged to each category (see Appendix S2); for example, flower‐rich habitat contained gardens, flowering crops, and urban parks, and tree cover contained woodland, hedgerow, and free‐standing trees. The proportion of each of the eight categories at each radius was calculated by summing the total area of all land cover classes contained within a category and dividing by the total area of the circle.

### 2.6 Principal components analysis

The resulting eight land‐use variables are too numerous to use for statistical analysis and are likely to be highly collinear; for example, proportion open land is likely to be correlated with proportion agricultural land. Principal components analysis (PCA) is a statistical tool that reduces dimensionality in a set of correlated variables by identifying a primary set of independent axes (or “principal components”) that explain the majority of the variation in the explanatory variables (Ringnér, 2008). It is particularly well suited to land‐use data and is often used as a step to refine multiple correlated land‐use variables in land classification protocols (Hahs & McDonnell, 2006; Owen et al., 2006).

A separate PCA was performed for each of the four radii using the *prcomp* function in R version 3.2.1 (R Development Core Team, 2015). The principal components that together captured 85% of the variation were selected as the land‐use variables for further analysis. The eigenvector scores [the weighting of a variable on a principal component; scores that depart from zero indicate increasing importance of that variable to the component (Hahs & McDonnell, 2006)] for each of the eight initial land‐use categories were extracted. Variables with scores greater than 0.4 or less than −0.4 were considered to show a strong association with the principal component. The types of variables strongly associated with a principal component were used to interpret the axis likely to be represented by the component (see Table 1).

**Table 1:** Results of principal components analyses on proportion land‐use categories at each of four radii

Radius		PC1	PC2	PC3
750 m	Standard deviation	2.154	1.467	—
	Proportion of variance	0.580	0.269	—
	Cumulative proportion	0.580	0.849	—
	*Eigenvector scores*			—
	Proportion impervious surface	**0.440**	0.000	—
	Proportion flower‐rich habitat	0.147	**0.512**	—
	Proportion domestic infrastructure	**0.458**	0.037	—
	Proportion open land	−0.247	**0.560**	—
	Proportion tree cover	−0.156	**−0.578**	—
	Proportion agricultural land	**−0.415**	0.258	—
	Proportion gardens	0.349	0.142	—
	Proportion road	**0.441**	0.032	—
500 m	Standard deviation	2.133	1.463	—
	Proportion of variance	0.569	0.268	—
	Cumulative proportion	0.569	0.836	—
	*Eigenvector scores*			—
	Proportion impervious surface	**0.442**	−0.054	—
	Proportion flower‐rich habitat	0.066	**−0.515**	—
	Proportion domestic infrastructure	**0.461**	−0.085	—
	Proportion open land	−0.289	**−0.515**	—
	Proportion tree cover	−0.085	**0.610**	—
	Proportion agricultural land	**−0.433**	−0.222	—
	Proportion gardens	0.338	−0.176	—
	Proportion road	**0.443**	−0.082	—
250 m	Standard deviation	2.141	1.440	—
	Proportion of variance	0.573	0.259	—
	Cumulative proportion	0.573	0.832	—
	*Eigenvector scores*			—
	Proportion impervious surface	**0.440**	−0.011	—
	Proportion flower‐rich habitat	0.157	**0.426**	—
	Proportion domestic infrastructure	**0.462**	0.033	—
	Proportion open land	−0.226	**0.583**	—
	Proportion tree cover	−0.139	**−0.599**	—
	Proportion agricultural land	**−0.418**	0.284	—
	Proportion gardens	0.373	0.188	—
	Proportion road	**0.429**	0.047	—
100 m	Standard deviation	2.019	1.407	1.007
	Proportion of variance	0.509	0.248	0.127
	Cumulative proportion	0.509	0.757	0.884
	*Eigenvector scores*			
	Proportion impervious surface	**0.424**	−0.115	**−0.428**
	Proportion flower‐rich habitat	0.174	**0.411**	**0.614**
	Proportion domestic infrastructure	**0.484**	−0.041	−0.151
	Proportion open land	−0.016	**0.673**	−0.165
	Proportion tree cover	−0.314	**−0.454**	0.416
	Proportion agricultural land	**−0.411**	0.367	−0.118
	Proportion gardens	**0.406**	0.121	**0.420**
	Proportion road	0.352	−0.093	0.163
*Interpretation*		Urban to rural	Open to covered	Flower‐rich to flower‐poor

The principal components (PCs) that together capture approximately 85% of the variation were selected for subsequent analysis. Eigenvector scores for each of the land‐use variables at each PC are shown and scores greater than 0.4 or less than −0.4 highlighted in bold and interpreted as having a strong relationship to that PC. The axes of each PC were interpreted based on these associated variables.

### 2.7 Cluster analysis

It is possible to use the principal components themselves as a final land‐use variable for subsequent analysis of the effect of land use on the response variables. This is appropriate if continuous variables are desired, and if the data suggest an evenly distributed, linear land‐use gradient. However, if a clustered land‐use structure is suspected, as in the present data (see Figure 5), an additional step of cluster analysis is recommended (Owen et al., 2006). This also has the advantage of combining all of the principal components into a single categorical land‐use variable, which can simplify analyses involving several covariates.

We performed a separate cluster analysis on the principal components for each radius [*hclust* function; R package *cluster* (Maechler, Rousseeuw, Struyf, Hubert, & Hornik, 2015)]. Hierarchical agglomerative clustering is a technique that examines distances between observations in the *n*‐dimensional space occupied by the principal components and sequentially pairs together the two closest observations (and later clusters) to form a new cluster (Zepeda‐Mendoza & Resendis‐Antonio, 2013). The exact outcome of the clustering depends on the method used to determine the distance between an observation and an existing cluster (e.g., taking the mean of the distance of all observations within a cluster as opposed to the minimum or maximum); here, we use Ward's method, which tends to produce clusters with more equal size (Ward, 1963). Similar land classification methods typically select optimum numbers of clusters using an ad hoc minimum group size based on practicality and geographical relevance (Bunce, Barr, Clarke, Howard, & Lane, 1996; Hall & Arnberg, 2002; Owen et al., 2006); following this approach, we split clusters so that each group contained a minimum of five sites. This produced a single categorical land‐use variable at each of the four radii (hereafter called R750, R500, R250, and R100).

### 2.8 Radius selection

As previously mentioned, the spatial scale at which an animal responds to the surrounding land use depends on numerous factors and cannot necessarily be determined a priori (Steffan‐Dewenter et al., 2002). A more data‐driven approach to determining spatial scale consists of conducting an initial analysis using the primary response variable or all response variables and using model selection to determine to which spatial scale the response variable(s) respond most strongly, and hence which land‐use radius to use for subsequent analysis.

We employed a model selection approach using Akaike's information criterion corrected (AICc) for small sample sizes. We built a full model for each of the four radii containing all covariates (in this case, weather and time covariates) and the relevant land‐use variable (R750, R500, R250, or R100) against the primary dependent variable (in this case, *peak colony size*). The land‐use variable contained in the model with the lowest AICc value (Johnson & Omland, 2004) was selected as the spatial scale to which the response variable responds most strongly and thus used for subsequent full analysis. If the best two or more models are within <2ΔAICc of each other, biological relevance (e.g., known foraging range) may be used to select the final radius from the best set. Alternative approaches for datasets with more than one dependent variable include performing this initial analysis for all dependent variables and selecting the spatial scale most commonly supported, or selecting the relevant spatial scale for each dependent variable.

The final categorical land‐use variable at the appropriate spatial scale may now be included in a full analysis using standard statistical methods.

## 3 RESULTS

### 3.1 Land cover map

The manual land cover classification step using satellite imagery in QGIS produced land cover maps for the area surrounding each of the 38 sites at a 750 m radius ( ;4). The most common land cover class was woodland, making up 21.2% of the total land area in the landscape surrounding the sites at a 750 m radius, followed by roads at 14.4% and housing at 12.1%. Site maps consisted of 36 to 845 (mean: 368) land cover patches.

**Figure 4:**
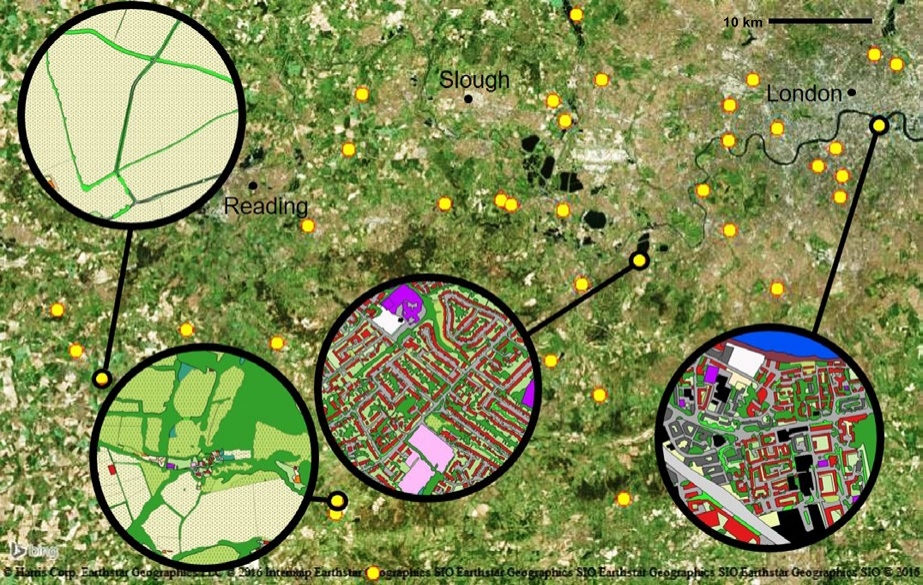
Land cover maps at a 750 m radius (inset circles) were generated for 38 sites in South‐East England; four representative sites across a gradient of urbanization are shown (large inset circles). Yellow circles indicate locations of sites

### 3.2 Principal components analysis

The results of PCAs carried out on the proportion of eight land‐use categories at each of the four radii are shown in Table 1. Approximately 85% of the variance was captured by two principal components (PCs) at the 750, 500, and 250 m radii and three PCs at the 100 m radius. By examining the eigenvalues of the land‐use categories in each of the PCs, the PCs were interpreted for all radii as PC1: urban to rural axis; PC2: covered to open axis and (at the 100 m radius only); PC3: flower‐rich to flower‐poor axis.

**Figure 5:**
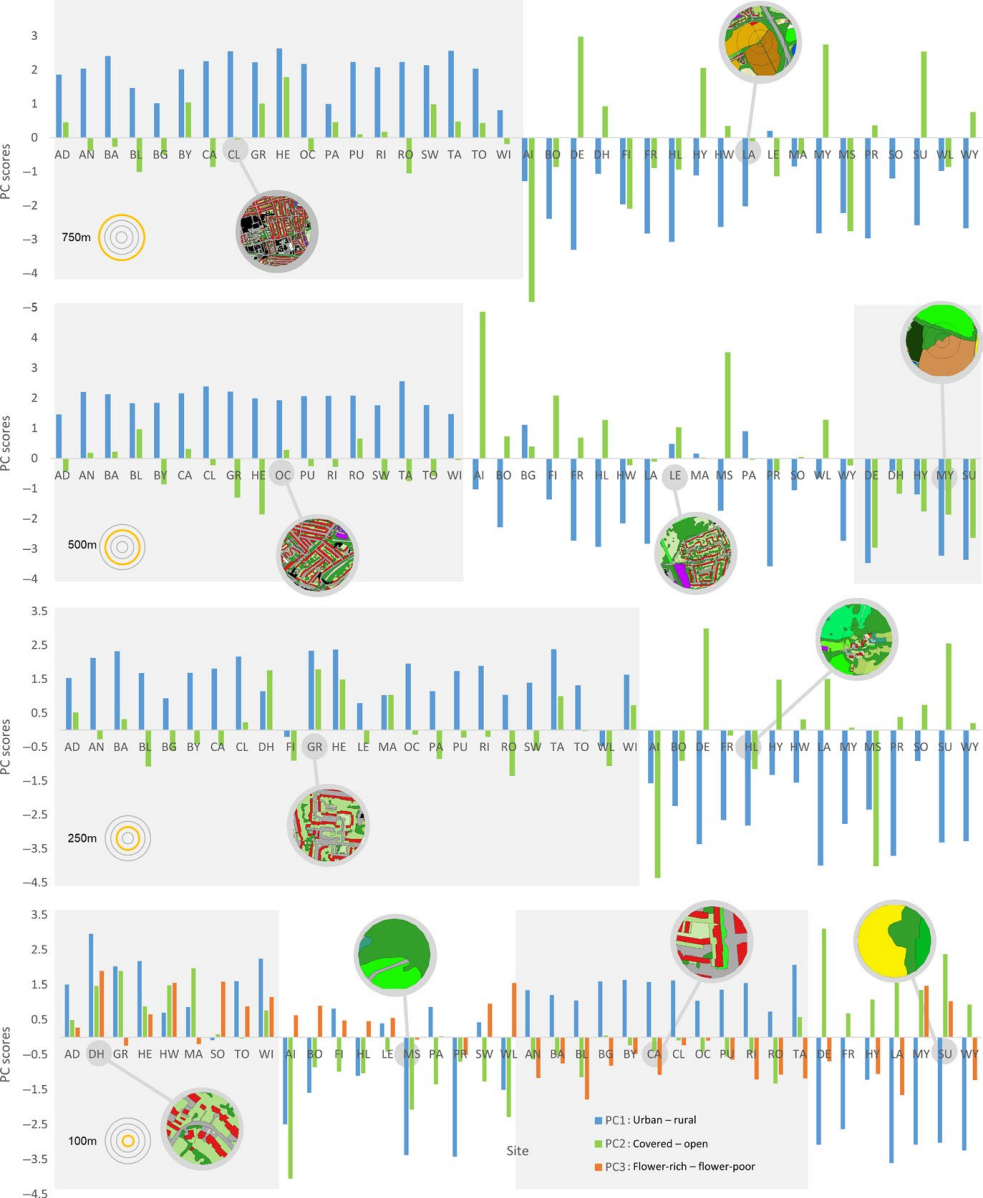
Eigenvector scores on principal components that captured approximately 85% of the variation in a principal component analysis (PCA) performed on land‐use variables classified at each of four radii around each study site (two‐letter codes). The clustering of the land‐use types generated from subsequent cluster analysis (Figure 6) is illustrated in the grouping of PC scores, shown here separated by shaded boxes (determined by the later cluster analysis). For example, at the 500 m radius (b), the “City” cluster (far left) is typified by a positive score on PC1 and neutral score on PC2, “Village” by positive to neutral PC1 and negative PC2, and “Agricultural” (far right) by negative PC1 and PC2. Inset circles show land cover maps at the relevant radius for representative sites for each group

The sites varied considerably in scores for each of the PCs at each radius (Figure 5), and the grouping of scores when all PCs were considered indicated clustering of the sites. For example, at the 500 m radius, a group with positive scores in PC1 and near‐zero scores in PC2, a group with negative in PC1 and positive in PC2, and a group with negative scores in both PCs were indicated (Figure 5b). As PC1 was interpreted as “urbanness” and PC2 as “openness,” this suggested a group that was built‐up and moderately open, a less built‐up and less open group, and a less built‐up but open group. This supported the employment of a formal cluster analysis.

### 3.3 Cluster analysis

**Figure 6:**
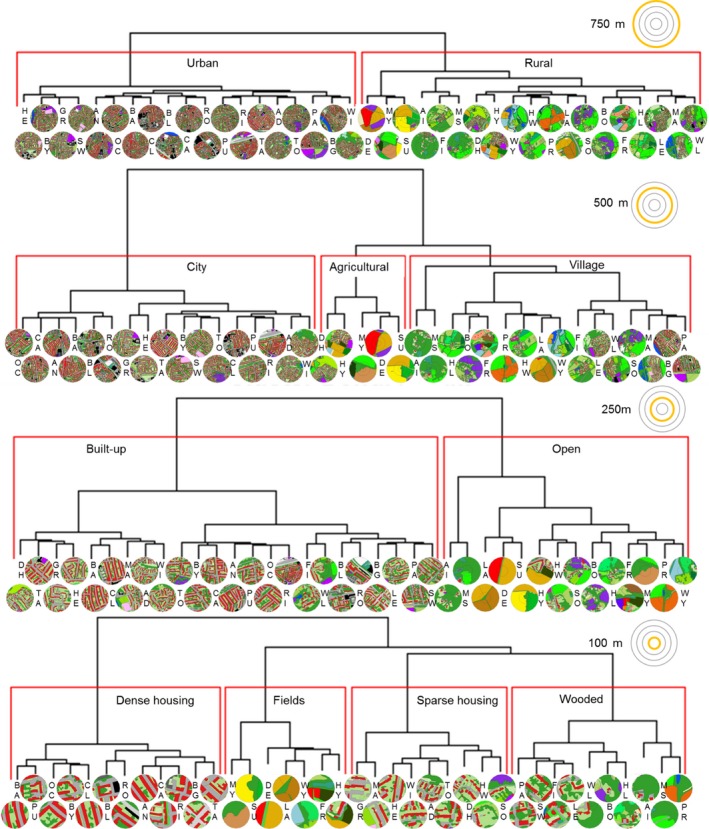
Cluster dendrograms of land use of 38 sites at a 750, 500, 250 and 100 m radii. Cluster analyses using Ward's method were performed on a set of principal components describing land use to group sites into categorical land‐use types (red boxes), which were given descriptive names from the landscape to the local scale. At the terminus of each branch, the two‐letter site name is given with an image of the GIS land cover map (see Appendix S1 for color legend)

Hierarchical cluster analysis using Ward's method on the principal components, with a minimum cluster size set to five, produced one categorical land‐use variable for each site, with two clusters at R750, three at R500, two at R250, and four at R100 (Figure 6). These were given descriptive names based on dominant land cover features of the sites in each cluster, ranging from the landscape to the local scale as follows: R750: urban, rural; R500: city, village, agricultural; R250: built‐up, open; R100: dense housing, sparse housing, wooded, fields.

### 3.4 Radius selection

Model selection of full models for each radius containing all covariates and the relevant categorical land‐use variable (R750, R500, R250 or R100) against the primary dependent variable *peak colony size* showed the model containing R500 to have the lowest AICc (ΔAICc to next best model: 2.7; Table 2). This suggests that *peak colony size* responds most strongly to land use at a 500 m radius surrounding the sites, and thus, that land use at the 500 m radius should be used in subsequent analysis.

**Table 2:** Model selection table to compare the response of the primary dependent variable, *peak colony size* to land use at four different radii (750, 500, 250, and 100 m) surrounding sites

Model	AICc	ΔAICc	*w*_*i*_
500 m land use + all covariates	93.3	0.00	0.65
750 m land use + all covariates	96.0	2.70	0.17
250 m land use + all covariates	96.8	3.52	0.11
100 m land use + all covariates	97.6	4.34	0.07

“All covariates” refers to temperature, humidity, and rainfall. Models are presented in order of ΔAICc from the best model alongside their respective Akaike weights (*w*_*i*_).

## 4 DISCUSSION

We describe a protocol for comprehensive classification of land use surrounding study sites suitable for pollinator ecology research. Our method employs a multistage approach that allows flexibility in adapting the specific steps to the data or research question involved. We start by manually mapping land cover using visual identification of land cover patches from satellite imagery at a resolution appropriate to the taxon studied. This is supplemented by ground surveys for land cover patches where habitats or resources are transient. Land‐use classes are then defined with specific reference to how the taxon interacts with the landscape based on knowledge of pollinator ecology, and the dimensionality of these variables is reduced using PCA. If the data suggest clustering of land‐use types, cluster analysis is performed to incorporate the principal components into a categorical variable. Finally, model selection is carried out to determine the appropriate spatial scale for further analysis. The final land‐use variable is a simple categorical variable at a single spatial scale, which contains information from multiple steps of refinement to generate a robust land classification from the pollinator's perspective.

In the case study presented here, the sites were selected to represent a gradient of urbanization. Interestingly, the land classification elucidated that, rather than forming a linear gradient, land‐use types clustered into relatively discrete categories that were not apparent from initial qualitative inspection. For example, at the 500 m radius, sites were clustered into three distinct groups: agricultural, village, and city. Land surrounding agricultural sites was dominated by fields, while village sites were characterized by housing in the immediate vicinity of the colony within a rural landscape, typically with extensive tree cover, and city sites consisted of dense innercity urban land. Without this approach, village sites may have not been distinguished from agricultural sites as both groups are predominantly surrounded by rural land. Our analysis identified the importance of the covered to open axis (PC2) in addition to the urban to rural axis and showed that agricultural and village sites differed sufficiently in how open they were to group separately following cluster analysis. Incidentally, analysis of the primary dependent variable in the dataset used in this case study, peak colony size, showed that colonies in the two land‐use types containing built‐up areas—village and city—grouped together, performing differently to agricultural colonies (Samuelson et al., submitted), supporting the land‐use clustering generated by our land classification protocol. To compare our classification with a commonly used variable in single‐step classification, we calculated percentage impervious surface for our sites: City sites contained mean 56.2% (±*SE*: 4.0%) impervious surface cover, while village and agricultural sites contained 13.8 (±3.7)% and 8.6 (±4.5)% impervious surface, respectively. This suggests our classification broadly agrees with the gradient described by this variable.

The primary value of our approach is in its flexibility to adapt to the focal study system and research question, due to the nature of building a land classification from the bottom‐up rather than co‐opting existing classifications (Owen et al., 2006). The ability to differentiate land cover patches at relatively fine resolution (up to 1:2,300 scale using the Bing Aerial QGIS layer) is more appropriate to pollinator spatial scales than many existing land cover maps [e.g., CORINE in Europe; scale: 1:100,000; minimum width of linear elements: 100 m (Büttner et al., 2004)]. Finer resolution data layers are available only for certain geographical areas (Troy & Wilson, 2006) and are often expensive, so our protocol may represent a good option for lower budget studies as the software used is open source. Existing land classifications that do contain data at a resolution relevant to pollinators such as the CEH Countryside Survey Land Cover Maps (e.g., LCM2015; Rowland et al., 2017) for the UK often combine land cover types that are extremely different from a pollinator perspective [e.g., “built‐up areas and gardens” in LCM2015 covers as varied land types as industrial estates, urban parkland, and domestic gardens (Rowland et al., 2017)], which can be separated using our approach. An additional advantage comprises the accommodation of transient habitats and resources through combining satellite imagery with ground survey, with initial land classification allowing targeted ground surveys of only the necessary land‐use patches. This may also be used to track seasonal differences in foraging resources, by supplementing a base map with regular ground surveys. There may also be value in further manipulation of the data generated from the land classification presented here. Many pollinator ecology studies are interested in landscape metrics such as landscape diversity (Boscolo, Tokumoto, Ferreira, Ribeiro, & dos Santos, 2017), which can be calculated from these data (Yeh & Huang, 2009), or individual land‐use elements such as the proportion area of a specific crop can be extracted for follow‐up questions. The method also allows qualitative uses of the land cover map, such as identifying foraging hot spots in honeybee waggle dance studies (Couvillon, Schürch, & Ratnieks, 2014). Finally, refining the classification using statistical tools rather than directly using individual land cover variables in subsequent analysis allows the incorporation of an extensive set of land‐use information within a single variable.

The most obvious limitation of our protocol is that it is time‐consuming. The most labor‐intensive step is generation of the land cover map. The time required depends on the complexity of the landscape and the resolution and upper radius selected; for example, it took an experienced researcher c. 4 hr to generate a map and classify the land cover patches for each site in our case study, which was at relatively high resolution in complex urban landscapes. Radius and resolution should therefore be selected at the minimum required for ecological relevance. Although the advantages of this step have been outlined above, where necessary, existing data layers may be used if they are available and relevant to pollinator ecology, and the later land‐use category definition, PCA, and cluster analysis steps applied to these data. These latter steps remain important because land‐use categories from existing data layers present the same problems with collinearity as study‐specific maps generated using the protocol described here. Ground truthing can also be time‐consuming, depending on landscape complexity and patch accessibility (here 2–3 hr per site). Ground truthing time can be minimized by targeting only relevant patches (e.g., arable fields) or using UAV (drone) surveys if the crop of interest is identifiable from a distance (e.g., oilseed rape).

Another limitation relates to errors introduced in classifying land cover patches from satellite imagery, particularly in cases where similar land cover types are hard to distinguish. This can be mitigated by verification with ground surveys and/or reanalysis of a subset of the data by an additional researcher to quantify error. Finally, manually drawing polygons to separate land cover patches can be subjective in terms of whether to separate or combine a patch. This highlights the importance of selecting a scale at which to view the satellite imagery at the start of the work, and it is important to note that “number of patches” is not an accurate measure of landscape heterogeneity for this reason.

The technique described here has potential applications in both pollinator ecology and other fields. While our case study is based upon *B. terrestris*, land use has been shown to impact numerous other pollinator taxa (Baldock et al., 2015; Senapathi et al., 2015), to which our approach may be applied. In pollinator ecology, our protocol may be combined with existing models to assess effects of resource availability with reference to land use (Kennedy et al., 2013; Lonsdorf et al., 2009; Williams et al., 2012) or with methods designed to evaluate landscape quality for pollinators (Couvillon & Ratnieks, 2015). Our method can also be applied to the studies of the interactions between land‐use and agricultural pest control, for which (as in pollinator ecology) land classification at a finer resolution than available data layers or with separation of specific land types (e.g., fallows, field margins) is often required (Bianchi, Booij, & Tscharntke, 2006), and varied responses of pest species and natural enemies to land use necessitate flexibility in the spatial scales of analysis (Thies, Roschewitz, & Tscharntke, 2005). Calculations of secondary landscape metrics that are known to affect natural pest control (Thies & Tscharntke, 1999), including landscape heterogeneity, may also be relevant to this field. Our protocol may be extended to other systems for which human‐focussed land classifications are not a suitable fit, as the spatial scale, land‐use categories, and resolution at which the land is classified may be adapted to the study system and research aims in question.

## 5 CONCLUSIONS

The aim of this study was to develop a land classification protocol for use in pollinator ecology research, from a pollinator rather than human perspective. Our protocol builds on the existing array of land classification techniques used in studies of land‐use effects on pollinators, expanding on methods employed in the pollinator literature (Banaszak‐Cibicka & Żmihorski, 2012; Sponsler & Johnson, 2015; Verboven et al., 2014) and adapting techniques developed in geographical research (Hahs & McDonnell, 2006; Owen et al., 2006). We have shown that bottom‐up land classification is feasible for studies such as that described in our case study and that useful land‐use data may be generated from doing so. Future research should expand on and refine this approach, and we suggest that land‐use studies in pollinator ecology should broadly adopt GIS‐based multistep land classification techniques.

## CONFLICT OF INTEREST

None declared.

## AUTHOR CONTRIBUTIONS

A.E.S. and E.L. conceived the study. A.E.S. developed the protocol, led the statistical analysis, and collected the empirical data used in the case study, with input and support from E.L. A.E.S. wrote the manuscript draft, and both authors produced the final edit.

## DATA ACCESSIBILITY

Data are available from the Dryad Digital Repository https://doi.org/10.5061/dryad.60s6123.
