# Supplementary material for: A land classification protocol for pollinator ecology research: An urbanization case study
Source: Ecol Evol. 2018 Apr 30;8(11):5598–610. doi: 10.1002/ece3.4087 (PMC6010921; doi:10.1002/ece3.4087)
Supplement: Supplementary file 1 [file ECE3-8-5598-s001.docx]

**A land classification protocol for pollinator ecology research: an urbanisation case study**

**Supporting Information**

Ash E. Samuelson* & Ellouise Leadbeater

School of Biological Sciences, Royal Holloway University of London, Egham, United Kingdom

*Corresponding author: [ash.samuelson.2014@live.rhul.ac.uk](mailto:ash.samuelson.2014@live.rhul.ac.uk)

Appendix S1

**List of land-use classes with assigned colours in GIS figures.**


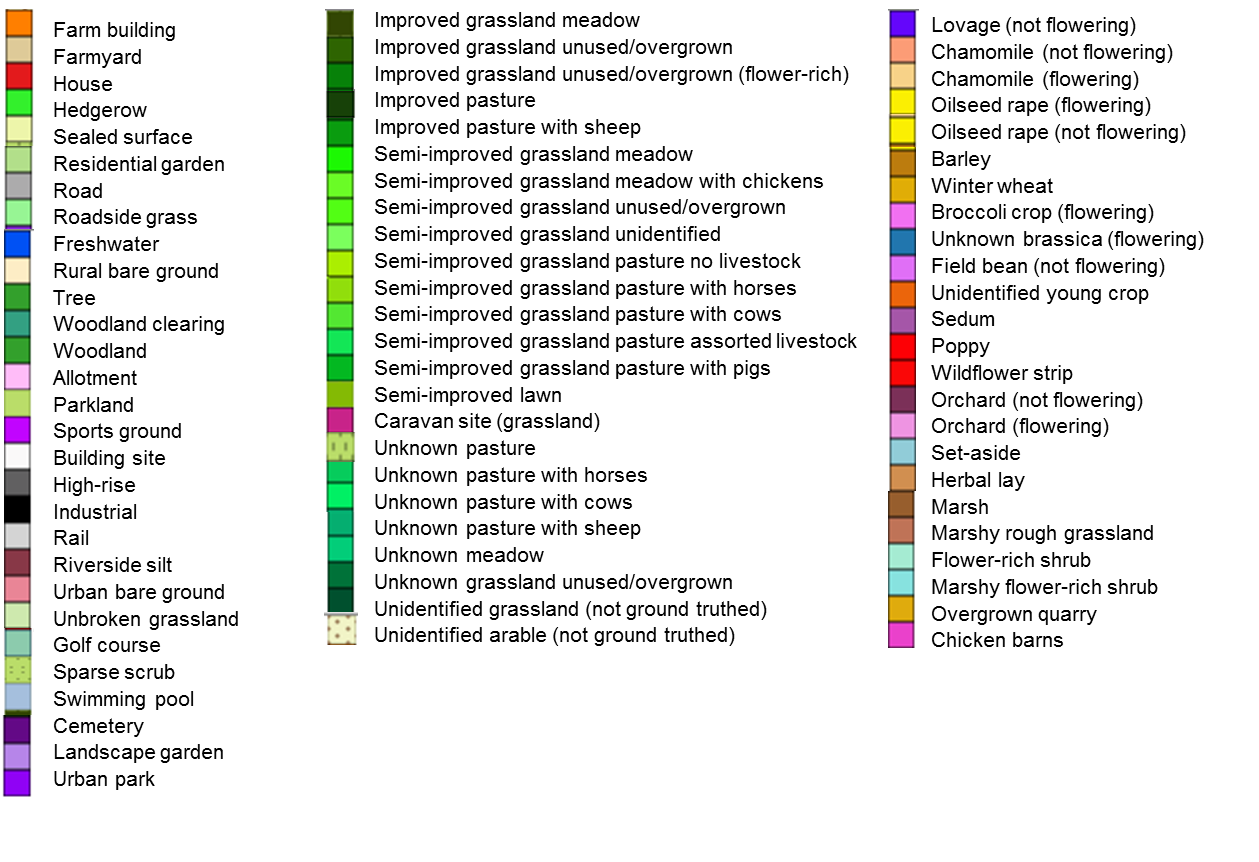


Appendix S2

**Land cover classes included in each of eight land-use categories**

| Code | Description | Impervious Surface | Flower-rich | Domestic Infrastructure | Garden | Tree Cover | Open | Agricultual | Road |
| --- | --- | --- | --- | --- | --- | --- | --- | --- | --- |
| FA | Farm building | 1 | 0 | 0 | 0 | 0 | 0 | 1 | 0 |
| FY | Farmyard | 0 | 0 | 0 | 0 | 0 | 1 | 1 | 0 |
| H | House | 1 | 0 | 1 | 0 | 0 | 0 | 0 | 0 |
| HR | Hedgerow | 0 | 1 | 0 | 0 | 1 | 0 | 0 | 0 |
| IS | Sealed surface | 1 | 0 | 1 | 0 | 0 | 1 | 0 | 0 |
| RG | Residential garden | 0 | 1 | 1 | 1 | 0 | 1 | 0 | 0 |
| RO | Road | 1 | 0 | 1 | 0 | 0 | 1 | 0 | 1 |
| ROG | Roadside grass | 0 | 0 | 1 | 0 | 0 | 1 | 0 | 0 |
| FW | Freshwater | 0 | 0 | 0 | 0 | 0 | 1 | 0 | 0 |
| RBG | Rural bare ground | 0 | 0 | 0 | 0 | 0 | 1 | 0 | 0 |
| T | Tree | 0 | 0 | 0 | 0 | 1 | 0 | 0 | 0 |
| WC | Woodland clearing | 0 | 0 | 0 | 0 | 0 | 1 | 0 | 0 |
| WL | Woodland | 0 | 0 | 0 | 0 | 1 | 0 | 0 | 0 |
| AL | Allotment | 0 | 1 | 1 | 0 | 0 | 1 | 0 | 0 |
| PL | Parkland | 0 | 0 | 0 | 0 | 0 | 1 | 0 | 0 |
| SG | Sports ground | 0 | 0 | 1 | 0 | 0 | 1 | 0 | 0 |
| BS | Building site | 1 | 0 | 1 | 0 | 0 | 0 | 0 | 0 |
| HI | High rise | 1 | 0 | 1 | 0 | 0 | 0 | 0 | 0 |
| IN | Industrial | 1 | 0 | 1 | 0 | 0 | 0 | 0 | 0 |
| R | Rail | 1 | 0 | 1 | 0 | 0 | 1 | 0 | 0 |
| TRS | Riverside silt | 0 | 0 | 1 | 0 | 0 | 1 | 0 | 0 |
| UBG | Urban bare ground | 0 | 0 | 1 | 0 | 0 | 1 | 0 | 0 |
| UG | Unbroken grassland | 0 | 0 | 1 | 0 | 0 | 1 | 0 | 0 |
| GC | Golf course | 0 | 0 | 0 | 0 | 0 | 1 | 0 | 0 |
| SS | Sparse scrub | 0 | 0 | 0 | 0 | 0 | 1 | 0 | 0 |
| SW | Swimming pool | 0 | 0 | 1 | 0 | 0 | 1 | 0 | 0 |
| LG | Landscape garden | 0 | 1 | 0 | 1 | 0 | 1 | 0 | 0 |
| GH | Cemetery | 0 | 0 | 1 | 0 | 0 | 1 | 0 | 0 |
| RHG | Manicured turf | 0 | 0 | 1 | 1 | 0 | 1 | 0 | 0 |
| UP | Urban Park | 0 | 1 | 1 | 0 | 0 | 1 | 0 | 0 |
| IGM | Improved grassland meadow | 0 | 0 | 0 | 0 | 0 | 1 | 1 | 0 |
| IGU | Improved grassland unused/overgrown | 0 | 1 | 0 | 0 | 0 | 1 | 1 | 0 |
| IGUF | Improved grassland unused/overgrown (flower-rich) | 0 | 1 | 0 | 0 | 0 | 1 | 1 | 0 |
| IP | Improved pasture | 0 | 0 | 0 | 0 | 0 | 1 | 1 | 0 |
| IPS | Improved pasture with sheep | 0 | 0 | 0 | 0 | 0 | 1 | 1 | 0 |
| SIGM | Semi-improved grassland meadow | 0 | 0 | 0 | 0 | 0 | 1 | 1 | 0 |
| SIGMC | Semi-Improved grassland meadow with chickens | 0 | 0 | 0 | 0 | 0 | 1 | 1 | 0 |
| SIGMH | Semi-Improved grassland meadow with horses | 0 | 0 | 0 | 0 | 0 | 1 | 1 | 0 |
| SIGU | Semi-Improved grassland unused/overgrown | 0 | 1 | 0 | 0 | 0 | 1 | 1 | 0 |
| SIG | semi-improved grassland (unidentified) | 0 | 0 | 0 | 0 | 0 | 1 | 1 | 0 |
| SIP | Semi-improved pasture no livestock | 0 | 0 | 0 | 0 | 0 | 1 | 1 | 0 |
| SIPH | Semi-improved pasture with horses | 0 | 0 | 0 | 0 | 0 | 1 | 1 | 0 |
| SIPC | Semi-improved pasture cows | 0 | 0 | 0 | 0 | 0 | 1 | 1 | 0 |
| SIPBS | Semi-improved pasture assorted livestock | 0 | 0 | 0 | 0 | 0 | 1 | 1 | 0 |
| SIPP | Semi-improved pasture pigs | 0 | 0 | 0 | 0 | 0 | 1 | 1 | 0 |
| SIL | Semi-improved lawn | 0 | 0 | 0 | 0 | 0 | 1 | 1 | 0 |
| CG | Caravan site (grassland) | 0 | 0 | 0 | 0 | 0 | 1 | 1 | 0 |
| UPA | Unknown pasture | 0 | 0 | 0 | 0 | 0 | 1 | 1 | 0 |
| UPH | Unkown pasture horses | 0 | 0 | 0 | 0 | 0 | 1 | 1 | 0 |
| UPC | Unkown pasture cows | 0 | 0 | 0 | 0 | 0 | 1 | 1 | 0 |
| UPS | Unkown pasture sheep | 0 | 0 | 0 | 0 | 0 | 1 | 1 | 0 |
| UM | unknown meadow | 0 | 0 | 0 | 0 | 0 | 1 | 1 | 0 |
| UGU | Unkown grassland unused/overgrown | 0 | 0 | 0 | 0 | 0 | 1 | 1 | 0 |
| PF | Unidentified grassland (not ground truthed) | 0 | 0 | 0 | 0 | 0 | 1 | 1 | 0 |
| AF | Unidentified arable (not ground truthed) | 0 | 0 | 0 | 0 | 0 | 1 | 1 | 0 |
| LVNF | Lovage (not flowering) | 0 | 0 | 0 | 0 | 0 | 1 | 1 | 0 |
| CHNF | Chamomile (not flowering) | 0 | 0 | 0 | 0 | 0 | 1 | 1 | 0 |
| CHF | Chamomile (flowering) | 0 | 1 | 0 | 0 | 0 | 1 | 1 | 0 |
| OSR | Oilseed rape (flowering) | 0 | 1 | 0 | 0 | 0 | 1 | 1 | 0 |
| BA | Barley | 0 | 0 | 0 | 0 | 0 | 1 | 1 | 0 |
| WW | Winter wheat | 0 | 0 | 0 | 0 | 0 | 1 | 1 | 0 |
| BRF | Broccoli (flowering) | 0 | 1 | 0 | 0 | 0 | 1 | 1 | 0 |
| UBF | Unknown brassica (flowering) | 0 | 1 | 0 | 0 | 0 | 1 | 1 | 0 |
| BNF | Beans (not flowering) | 0 | 0 | 0 | 0 | 0 | 1 | 1 | 0 |
| YUC | Unidentified young crop | 0 | 0 | 0 | 0 | 0 | 1 | 1 | 0 |
| SE | Sedum | 0 | 1 | 0 | 0 | 0 | 1 | 1 | 0 |
| PO | Poppy | 0 | 1 | 0 | 0 | 0 | 1 | 1 | 0 |
| WS | wildflower strip | 0 | 1 | 0 | 0 | 0 | 1 | 1 | 0 |
| ORNF | Orchard not flowering | 0 | 0 | 0 | 0 | 0 | 1 | 1 | 0 |
| ORF | Orchard flowering | 0 | 1 | 0 | 0 | 0 | 1 | 1 | 0 |
| SA | Set aside | 0 | 0 | 0 | 0 | 0 | 1 | 1 | 0 |
| HL | Herbal lay | 0 | 1 | 0 | 0 | 0 | 1 | 1 | 0 |
| MA | Marsh | 0 | 0 | 0 | 0 | 0 | 1 | 0 | 0 |
| RMA | Marshy rough grassland | 0 | 0 | 0 | 0 | 0 | 1 | 0 | 0 |
| FRS | Flower-rich shrub | 0 | 1 | 0 | 0 | 0 | 1 | 0 | 0 |
| MFRS | Marshy flower-rich shrub | 0 | 1 | 0 | 0 | 0 | 1 | 0 | 0 |
| OQ | Overgrown quarry | 0 | 1 | 0 | 0 | 0 | 1 | 0 | 0 |
| CB | Chicken barns | 0 | 0 | 0 | 0 | 0 | 0 | 1 | 0 |
